# Phenome of pearl quality traits in the mollusc transplant model *Pinctada margaritifera*

**DOI:** 10.1038/s41598-018-20564-1

**Published:** 2018-02-01

**Authors:** Chin-Long Ky, Virgile Quillien, Floriane Broustal, Claude Soyez, Dominique Devaux

**Affiliations:** 1 Ifremer, UMR 241, EIO, Labex Corail, Centre du Pacifique, BP 49, 98719 Taravao, Tahiti Polynésie Française; 2 SCA Regahiga Pearls, BP 48, 98755 Rikitea, Gambier Polynésie Française

## Abstract

The bivalve *Pinctada margaritifera* exhibits three main transplant phenotypes derived from the donor (from which a mantle graft tissue, the *saibo*, is excised), the recipient (into which the *saibo* is implanted with a nucleus, leading to the formation of a pearl sac “chimera”) and the cultured pearls themselves. This first phenome study on the species derived from a large experimental graft. Transplant phenotype was assessed at three scales: 1) macro, pearl size, colour, grade, 2) micro, pearl surface microstructure, and 3) molecular, biomineralisation gene expression level in saibo and pearl sac tissues. From donor to pearl, the phenome revealed fine variations of quality traits dependent on the position on the mantle where the *saibo* was cut, whose variation could overlap with inter-individual donor phenotype differences. A single donor phenotype could therefore produce multiple pearl phenotypes at the scale of the *saibo* position, mirroring its original activity at the mantle position level and the colour and shape of the shell. This phenome study provides essential information on phenotypic trait architecture enabling us to explore and explain the main biological functions and pave the way for a phenomic project on *P. margaritifera* that could benefit the pearl industry.

## Introduction

The phenotype is the result of the interplay of genetics with developmental, environmental and stochastic influences, where the intensity, frequency, order and interaction of these influences all affect the outcome. In the era of next generation sequencing with continued decreases in cost and increasing availability of high-throughput genotyping platforms, genomic data acquisition and associated bioinformatics treatments have become common even for non-model organisms. The complexity of plant and animal genomes, constructed from a pool of four nucleic acids and organized in a one-dimensional sequence, pales in comparison to their corresponding phenome. Phenome serves an unknown number of functions, many of which show enormous inter-individual variation that is at best only partially understood and for which the dimensionality remains unknown. This is not only due to the recent advances in genomics but also the complex multidimensional nature of phenotypes^1^. The vast number of phenotypic states of a genotype can be viewed as its phenotypic space, which is often referred to as its phenome. In practice, the phenome is a theoretical entity which can never be fully characterized^2^. Phenomics, operationally defined as the systematic study of phenotypes, is critically important to provide essential information for advances in genetic improvement for many cultured plant and animal species in the post-genomic era.

Species from the *Pinctada* genus are regularly used for the production of valuable free round cultured pearls, the only gems produced by a living organism^3^. The production of cultured pearls is both unique and biologically complex compared to that of other aquaculture industries. These are nucleated pearls produced in the gonad of a recipient pearl oyster following surgical implantation of a spherical shell-based bead (the nucleus) together with a piece of mantle (the *saibo*) cut from a particular section (located between posterior and anterior zone, without considering the junction of the mantle with the oyster gills) of a selected donor oyster. The mantle is clearly a metabolically and transcriptionally active tissue, indispensable for mollusc shell formation, with prominent transcriptional activity of biomineralisation genes^4^. The biomineralisation process is responsible for both pearl and shell formations. A few weeks after the graft operation, the pearl sac develops as a result of the epithelial cells of the mantle epidermis growing around the nucleus to completely cover it^5^. The pearl formation process then starts, by the deposition of successive biomineral layers onto the nucleus^6^. *P. margaritifera* is an ideal model animal for the study of biomineralisation because of the intriguing microstructure of: 1) its shells, which consist of outer calcitic prismatic layers and inner aragonitic nacreous layers, and 2) the pearls, which display mostly aragonitic structures, similar to the inner layer of the shell in both appearance and structure, through the chimeric pearl sac activity. Cultured pearl value is based on four main quality traits: size (nacre weight and thickness), shape, colour (including darkness level) and grade (the combination of lustre and surface quality)^3,7^.

Phenotype transmission in the *Pinctada* transplant model has been mostly studied from an applied point of view, in relation to its economic importance in the pearl industry. Indeed, much research effort has been focused on size and colour determination in relation, to the donor oyster, the recipient oyster, their interplay, and their interaction with the environment^8–11^. In *P. margaritifera*, pearl size is known to be mostly driven by recipient oyster growth performance, and the donor oyster to be responsible for pearl qualitative trait determination, including colour^12^. The genotype of the donor therefore persists within the recipient in the form of the pearl sac. This chimeric organ displays more complex interactions, particularly when spatial (geographic origin of the population, depth of culture practices), temporal (age of hatchery-produced or wild collected spat), and environmental (grow-out site culture, season of graft, experimental temperature or pH variation) factors are introduced into the equation of the pearl quality trait determination^3^. Previous studies have all considered the transplant model at the individual scale: *i.e*., at the level of individual pearl oysters^13–15^. The associated phenotypic variations recorded were then systematically related to inter-individual/ family variation among donors or recipients.

**Figure 1:**
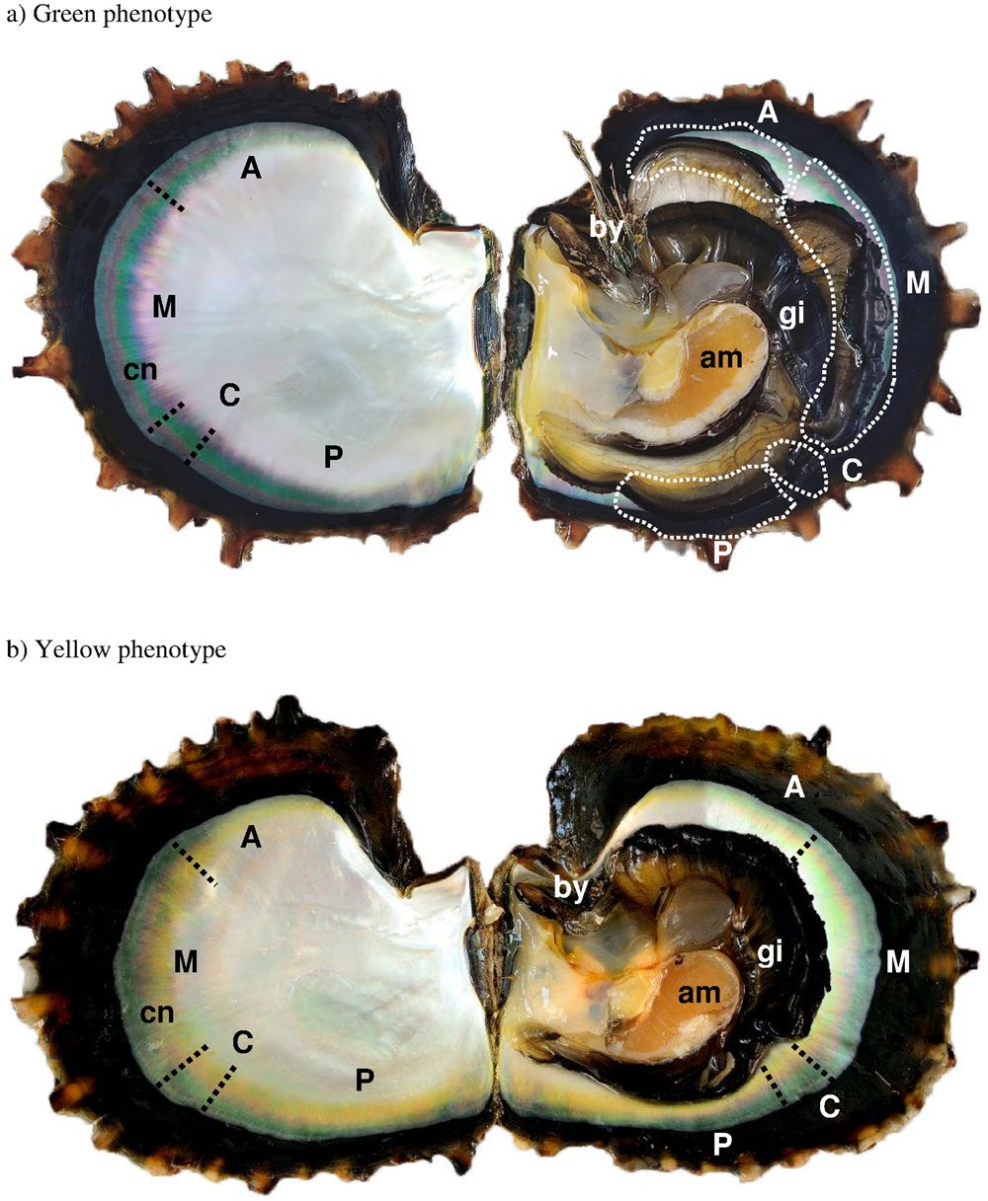
Donor *Pinctada margaritifera* of green (**a**) and yellow (**b**) phenotypes, each with two shell valves showing, on the right, the four sections of the entire dilated mantle tissue in (**a**), and contracted mantle tissue in (**b**): posterior position (P), connection with the gill (C), middle corresponding to the zone usually used for commercial *saibo* production (M) and anterior (A). In each case, on the left, the correspondence is shown with the zones of contact with the inner shell zone that exhibits the colourful band characteristic of donor oysters. The dotted lines indicate the areas of mantle tissue excised for *saibo* production from the four positions A, M, C and P. General anatomy: am, adductor muscle; gi, gills; by, byssus; cn, coloured nacreous zone.

The objective of the present study was to explore for the first time the phenome of cultured pearl quality traits at the scale of the *saibo*, *i.e*., at an intra-individual donor scale. For this, two fixed hatchery-produced pearl oyster phenotypes of *P. margaritifera*, exhibiting predominately green or yellow inner shell coloration (twelve of each) were used as donors. The entire mantle was taken for *saibo* production, including all the following four sections: posterior, connection (with gills), middle (used in commercial production) and anterior (Fig. 1). Experimental grafts (N = 1798) with traceability between donor line and *saibo* mantle position has been designed and performed in a single culture site. These grafts provided the biomineralised materials for the phenome study: the *saibo* tissue, the chimeric pearl sac tissue, and the associated pearls. Phenotypic data were collected at the macroscopic level (pearl quality traits), microscopic level (pearl surface ultrastructure) and molecular scale (expression level of a panel of biomineralisation genes representative of the nacreous aragonite and/or prismatic calcite synthesis in both *saibo* and pearl sac, derived from each of the mantle sections). This first phenome study, initiated with a set of easy-to-use tools, will provide: 1) basic knowledge to help us to understand phenotypic transmission, range of variation in an animal transplant model, and 2) useful information for the improvement of pearl quality for the industry.

## Results

### Experimental graft

**Figure 2:**
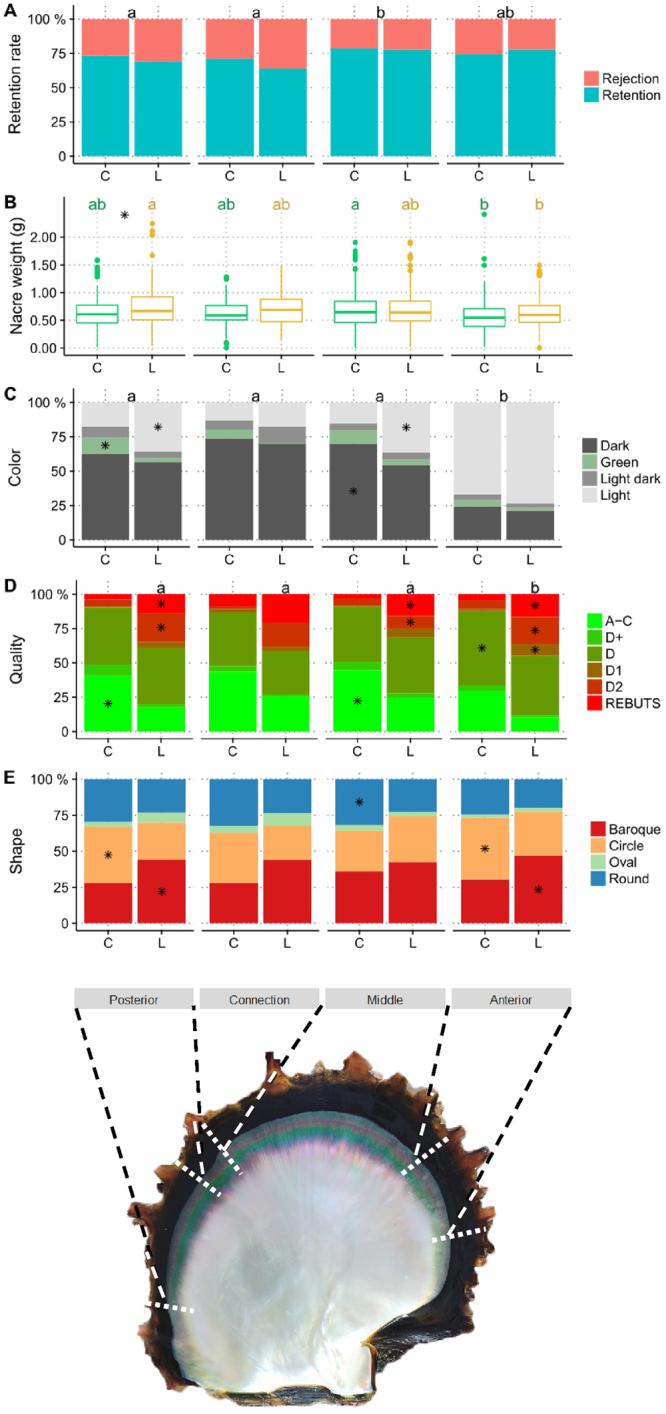
Graft and cultured pearl quality trait variation in *P. margaritifera* according to both mantle graft position (posterior, connection, middle and anterior) and the two phenotypes of donor (green C and yellow L). The five variables are the: (**A**) experimental graft retention rate; (**B**) cultured pearl nacre weight in g (boxplot); (**C**) pearl colour category percentages (dark, green, light dark and light), (**D**) pearl grade rate (A–C, D^+^, D, D1, D2 grade and *rebuts*) and (**E**) pearl shape rate (baroque, circle, oval or round). Letters indicate significance between the mantle graft positions within each variable. Asterisks indicate within each mantle graft position the significant differences between the proportions of the categories of each cultured pearl trait, between the C (green) and L (yellow) phenotypes. The shell valve corresponded to a green C phenotype donor oyster.

The nucleus retention rate was 75.1% (N = 1350) at 45 days post-grafting (the remainder, 24.9%, N = 448, correspond to nucleus rejection, oyster mortalities and/or predation). Between the two phenotypes used as donors (green and yellow), no significant difference was detected for overall retention rate, even at the mantle section scale (*i.e*., pairwise comparison between oysters grafted with the same mantle section between the two phenotypes). By contrast, comparison between the mantle sections revealed that the middle position had significantly a higher retention rate than the posterior (+7.0%; *p* = 0.0315) and connection positions (+10.7%; *p* = 0.0315) (Fig. 2A).

### Cultured pearl quality traits

The overall mean cultured pearl nacre weight was 0.67 g (±0.32 g). A significant difference (*p* = 0.031) was detected between the two donor phenotypes, with grafts from the yellow donor leading to heavier nacre (+6.2%), in comparison with the green phenotype: 0.65 (±0.31 g) *vs*. 0.69 (±0.33 g). This was due, at the mantle position scale, to the anterior position on the yellow phenotype, which led to nacre 18.8% heavier (*p* = 0.019) than the green phenotype (Fig. 2B). Differences between the mantle positions were observed within the donor phenotypes. For the green phenotype, the middle section was 17.3% heavier than the anterior one (0.68 ± 0.31 g *vs*. 0.58 ± 0.30 g; *p* = 0.001). For the yellow phenotype, the two extremities of the mantle gave the most disparate results: the posterior position was 20.6% significantly (*p* < 0.001), heavier than the anterior one (0.76 ± 0.39 g *vs*. 0.63 ± 0.27 g).

The overall distribution of the cultured pearls among the colour categories was as follows: 53.1% (N = 632) for dark, 5.8% (N = 70) for green, 5.4% (N = 64) for light dark and 35.7% (N = 425) for light. The anterior section was significantly different from the three others and showed the highest rate of light pearls whatever the donor phenotype considered. For the yellow phenotype, 73.5% light pearls were found with anterior section grafts compared with an average of 30.0% for the other sections (Fig. 2C). The same tendency was observed in the green phenotype, with 66.9% of light pearls from the anterior section grafts and an average of 15.3% for the three other sections. The posterior and middle sections differentiated the two donor phenotypes, whereas the connection and anterior sections showed no difference between the colour rates. The yellow phenotype posterior position grafts produced 18.1% more light pearls than those cut from the same position on green phenotype donors (*p* = 0.001), which showed a significantly higher proportion of green pearls (+8.9%; *p* = 0.014). The middle position also had a significantly higher proportion of light pearls for the yellow phenotype (+21.0%; *p* < 0.001), while the green phenotype showed more dark pearls for this position (+15.7%; *p* < 0.001) (Fig. 2C).

For the cultured pearl quality grade, no significant differences between positions were detected for the green phenotype (Fig. 2D). For the yellow phenotype, the anterior position was different, with only 10.5% top quality pearls (A–C grade) compared with 22.9% on average for the three other positions (*p* = 0.002). Comparison between the two phenotypes showed that: 1) the green phenotype produced significantly (*p* < 0.001) more pearls of grades A–C, with +23.1% and +20.3% for the posterior and middle positions, respectively, 2) the yellow phenotype produced significantly more *rebut* (reject) grade peals, with +9.5% (*p* = 0.012) and +12.1% (*p* < 0.001) for the posterior and the middle positions, respectively, and also more D2 grade pearls with + 16.0% (*p* < 0.001) and +9.8% (*p* = 0.041) for the posterior and middle positions, respectively (Fig. 2D). A significant difference was also observed between the two phenotypes when comparing the results of anterior position grafts, with the yellow donors producing more *rebuts* (+12.1%, *p* < 0.001), D2 (+13.3%, *p* < 0.001) and D1 (+7.1%, *p* < 0.001) pearls and less grade D pearls (−10.5%, *p* = 0.01), than the green phenotype (Fig. 2D).

For cultured pearl shapes (Fig. 2E), no significant differences were detected within donor phenotype groups among the four different mantle positions from which *saibo* was cut. By contrast, significant differences were observed between the two donor colour lines. For posterior and anterior positions, yellow phenotype donors produced significantly more baroque pearls than green phenotype donors with +16.2% (*p* = 0.01) and +16.6% (*p* = 0.006), respectively. For these two positions pearl circles were, on the contrary, more frequent in pearls from green phenotype donors than yellow ones, with +13.4% (*p* = 0.034) for posterior position *saibo* and +12.5% (*p* = 0.038) for the anterior position. For the middle position, round pearls were significantly more frequent with the green phenotype (+8.9%, *p* = 0.02).

### Cultured pearl surface ultrastructure observation

**Figure 3:**
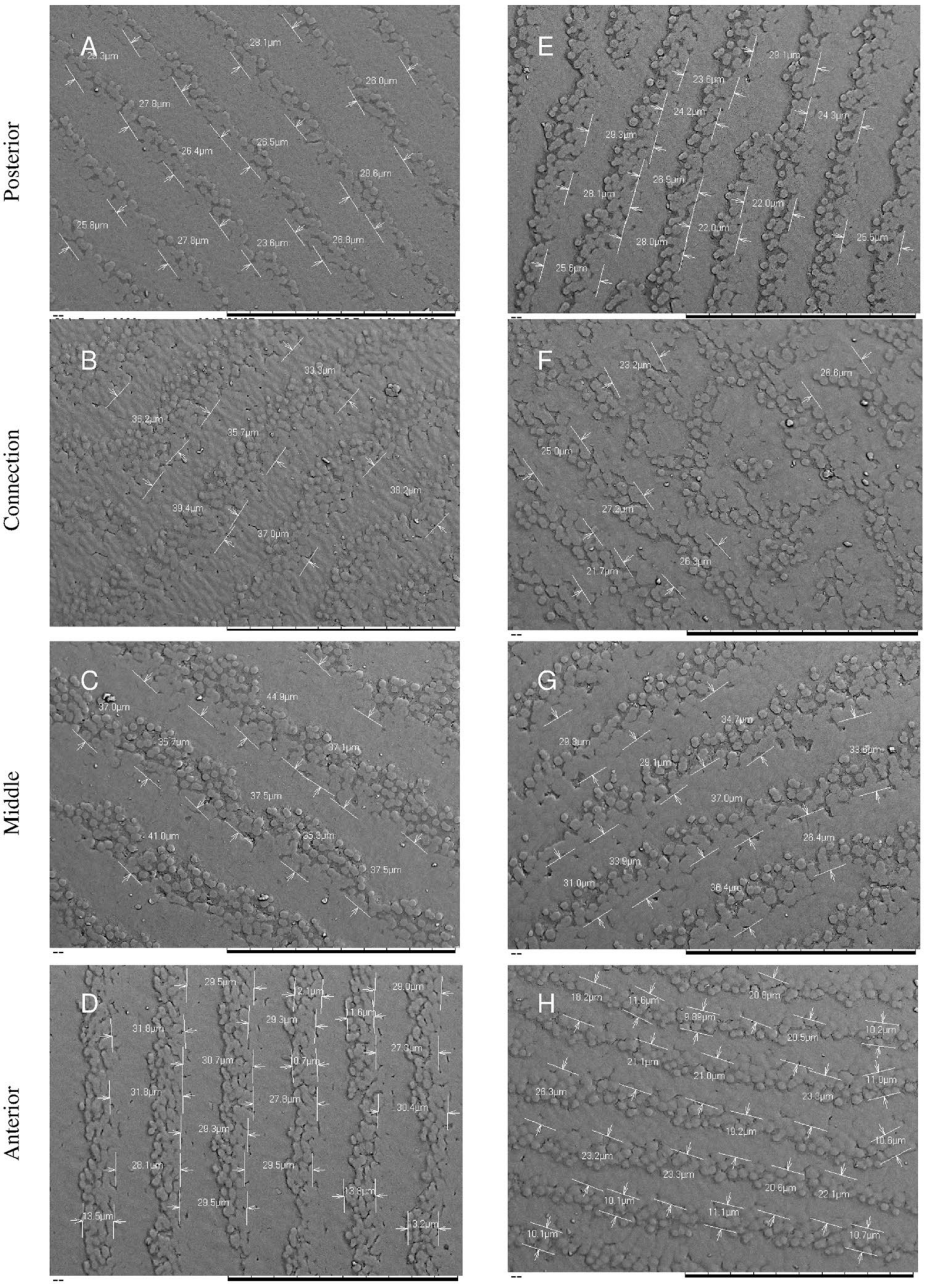
*P. margaritifera* cultured pearl surface, assessed by electronic microscopy (magnification: x1000) according to mantle graft position (posterior, connection, middle or anterior) based on grafts with tissue from two donor oysters. The first column (**A**,**B**,**C** and **D**) corresponds to sample of pearl surfaces from grafts made with the same yellow phenotype donor, whereas the second column (**E**,**F**,**G** and **H**) corresponded to pearl surface from another donor, which had the green phenotype. Cultured pearl grades were all D grade for those pearls produced with the yellow phenotype donor (first column) and (**A**–**C**) grade for pearls produced with the green phenotype donor (second column). The black bars at the bottom of each picture correspond to 100 µm. Distances between the fronts of aragonites were expressed in µm and illustrated with white bars and arrows.

The mineralised portion of the nacre was observed on the cultured pearl surface by scanning electron microscopy and was seen to consist of aragonite tablets organized into growth fronts (Fig. 3). Observation of the corresponding microscopic patterns showed clear differences in the distance between the parallel growth fronts on the pearl surface, between the different mantle sections. The growth fronts of pearls produced with middle position *saibo* were significantly larger (35.1 ± 4.5 µm; Fig. 3C,G), than those of the connection zone (30.8 ± 6.4 µm; *p* = 0.045; Fig. 3B,F) and the posterior and anterior sections, which showed similar growth front distances (mean of both: 26.0 ± 3.5 µm; *p* < 0.0001; Fig. 3A,E,D,H).

### Biomineralisation gene expression levels in *saibo*

**Figure 4:**
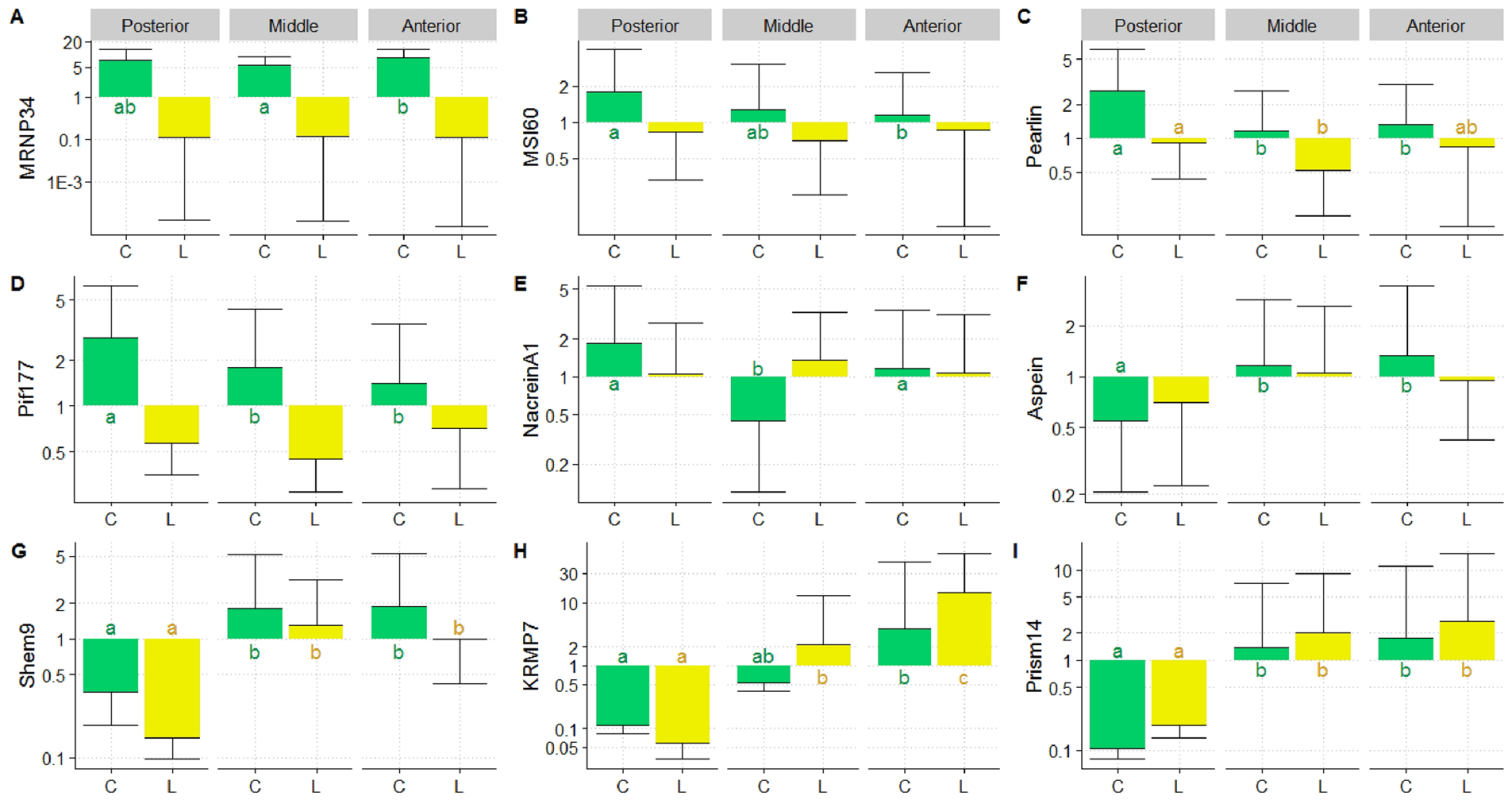
Relative expression of nine biomineralization genes in the mantle graft tissue (mixed of 3 *saibo* per position) of *P. margaritifera* obtained from the green C (Posterior N = 6; Middle N = 10; Anterior N = 11) and yellow L (Posterior N = 6; Middle N = 12; Anterior N = 6) donor phenotypes. Y axes are in the logarithmic scale. Error bars correspond to standard deviations. Letters indicate significance between mantle graft positions within each phenotype.

Concerning the genes involved in the nacreous layer produced from the mantle, all four corresponding genes (*MRNP34*, *MSI60*, *Pearlin* and *Pif177*) showed the same expression profile, which distinguished the yellow and the green donor phenotypes, whatever the mantle position considered (Fig. 4). The *saibo* originating from the green donor phenotype systematically showed overexpression of these four genes in comparison to the *saibo* from the yellow variant (Fig. 4A–D). For example, in *saibo* from the green phenotype the *Pif177* gene was overexpressed by 4.90 (*p* = 0.01), 3.98 (*p* < 0.001) and 1.96 (*p* = 0.023) times for the posterior, middle and anterior positions respectively. Comparison among mantle sections within the green phenotype revealed systematic overexpression of genes in the posterior section compared with the middle and anterior sections, except for *MRNP34*.

For the four genes involved in prismatic layer formation (*Aspein*, *Shem9*, *KRMP7* and *Prism14*), near identical expression profiles were observed among donor phenotypes and mantle positions (Fig. 4). The same tendencies were observed between the green and yellow donor phenotypes for all prismatic genes, except for the *KRMP7* gene expression in the middle section, where there was significant overexpression (×4) in the yellow phenotype compared with the green one (Fig. 4H). Expression of *Shem9* showed significant differences between: 1) phenotypes for the anterior position only (*p* = 0.048), with nearly twice the expression level in the green phenotype compared with the yellow, and 2) mantle section for both phenotypes only for the anterior position which was different from the two others (Fig. 4G). For *Aspein* gene expression, no differences were observed between the donor phenotypes for any of the mantle positions.

Expression levels of the *Nacrein A1* gene (involved in both nacreous and prismatic formation), were not significantly different between mantle positions for the yellow donor phenotype (Fig. 4E). By contrast, the middle section of the green phenotype was significantly different from the anterior and posterior positions. Inter-phenotype comparison revealed significant differences for the posterior and middle sections, with respectively overexpression factors of 1.77 for the green and 3.02 for the yellow phenotype.

### Biomineralisation gene expression levels in the pearl sac

**Figure 5:**
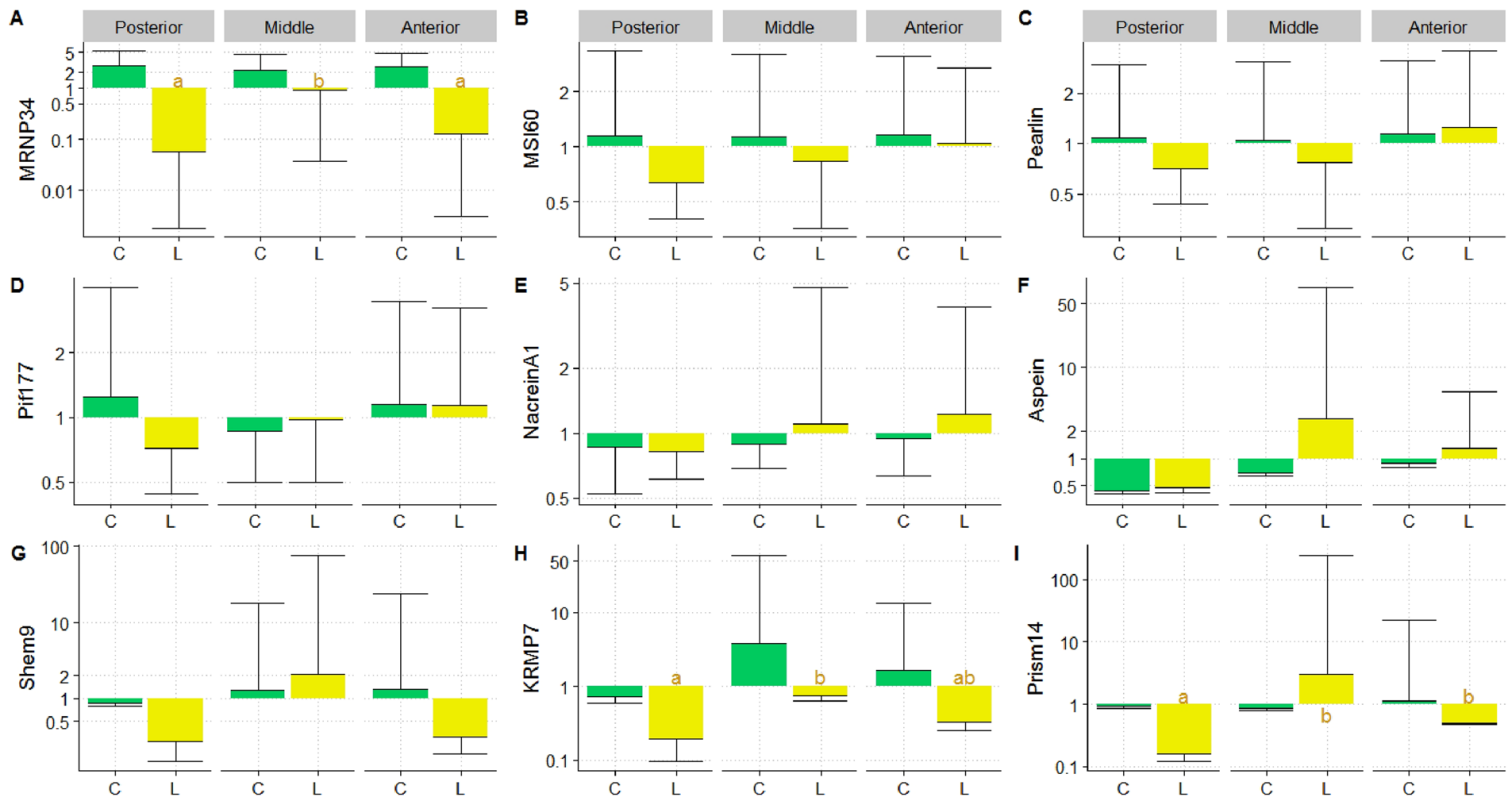
Relative expression of nine biomineralization genes in the pearl sac of *P. margaritifera* originated from the green C (Posterior N = 13; Middle N = 20; Anterior N = 32) and yellow L (Posterior N = 13; Middle N = 20; Anterior N = 16) donor phenotypes. Y axes are in the logarithmic scale. Error bars correspond to standard deviations. Letters indicate significance between the mantle graft positions within each phenotype.

For the genes involved in the nacreous layer produced by the pearl sac, the expression of *MSI60*, *Pearlin* and *Pif177* were not different between either phenotypes or mantle positions (Fig. 5B–D). For *MNRP34* gene expression, differences were detected only for the yellow phenotype, where the posterior (*p* = 0.002) and anterior positions (*p* = 0.006) were overexpressed 47 and 20 times, respectively, in comparison to the middle (Fig. 5A).

For the genes implicated in cultured pearl prismatic layer formation, the expression of the *Aspein* gene showed no significant difference between phenotypes or positions (Fig. 5F). *Shem9* was expressed 4.7 times more in pearl sacs from anterior position *saibo* from green donor phenotypes than in those from the same position in the yellow donor phenotype (*p* = 0.04) (Fig. 5G). For *KRMP7* gene expression, the two phenotypes were highly different for all mantle sections: in pearl sacs from the green phenotype donors, *KRMP7* was overexpressed 5.4 times for the anterior position, 4.5 times for the middle section and 3.8 for the posterior position, compared with the yellow phenotype (Fig. 5H). For *Prism14* gene expression, only the posterior position showed a significant difference between phenotypes, with the green donor phenotype showing significantly higher expression (5.9 times, *p* = 0.014). Within phenotype, no significant differences were revealed between mantle positions (Fig. 5I).

Concerning the gene involved in both nacreous and prismatic layer formation, *Nacrein A1* showed no significant differences between donor phenotypes or mantle positions (Fig. 5F). For the two phenotypes, no significant differences were detected between positions.

## Discussion

Phenotypic variations were assessed in the present study in a particular transplanted animal model, *P. margaritifera*, which combines a complex three-way interaction between the donor oyster, recipient oyster and the final cultured pearl product. The associated phenome, in relation to biomineralising and biomineralised tissues (graft tissue, pearl sac tissue, pearl samples) was assessed through a set of easy-to-use tools (visual and microscopic observation, RT-PCR) applied at three observation levels: macro-, micro- and molecular.

This initial cultured pearl quality phenome mirrored, to a certain extent, *P. margaritifera* shell morphology and characteristics. Oyster shell and cultured pearls are respectively formed in two distinct biomineralised tissues: the mantle and the pearl sac, which are derived from mantle tissue from the donor^16^. Around the nucleus, a pearl sac is formed by proliferation of the outer mantle epithelial cells of the mantle graft, which secretes successive nacre layers on the nucleus^17,18^. The pearl sac consists of mucous cells containing large acidophilic granules and epidermal cells^19^ that secrete proteins resulting in cultured pearl formation, a highly controlled biomineralisation process similar to the development of the inner shell regulated by the mantle^20^. Similarly to other bivalves, the shell of *P. margaritifera* consists of two polymorphs of calcium carbonate: the inner nacreous layer, which is composed of aragonite, and the outer prismatic layer, which is made of calcite^21–23^. Shell formation is a highly controlled process involving multiple matrix proteins^24–26^. In the *Pinctada* genus, the anterior zone of the shell is characterised by two growth-related features. First, the byssus location gives an anteriorly oblique shell conformation, with specifically subtriangular anterior auricles. Second, the concentric growth lines are closer in the anterior zone, than in the posterio-ventral part of the shell corresponding to the other mantle sections^3^.

At macro-scale observation, and for pearl size, results revealed that the anterior shell-growth potential was conveyed by the *saibo* from the donor into the chimera formed in the recipient and thereby impacted nacre weight and thickness of pearls produced, giving smaller pearls compared to those grown from *saibo* from the other mantle sections, whatever the donor phenotype considered. This intra-phenotype variation from the donor, at the level of mantle position, was sometimes greater than variation observed at an inter-phenotype level. This result is consistent with earlier findings where donor effect for pearl size was shown using the same middle mantle section compared among wild^27^ or hatchery-produced oysters^28^. The present study pointed the existence of a mantle effect within a same donor phenotype, which must be considered for pearl size determination to avoid any artefacts for genetic selection program aiming to improve this trait.

Colour transmission to the pearls is also dependent on the section of the mantle used at the scale of the individual donor. Here again, intra-individual variation could be greater than inter-individual variation. This was the case for example for the attractive green pearl colour, whose rate was significantly lower for the posterior section of yellow donor phenotypes compared with overall results from green donors, but similar to results from the green anterior section. Differences in pearl colour distribution also mirrored, to a certain extent, the inner shell colour profile of the donor oyster. Indeed, when looking at the interior nacreous surface of the shell, the anterior zone corresponds to the least colourful part of the donor oyster, compared with the parts adjacent to the other three sections, which show the largest and strongest intensity of the characteristic coloured band, particularly in the middle section of the mantle (Fig. 1). It is commonly known that donor tissue influences the colour of the resulting pearls and is mostly dependent on the species of *Pinctada* used^11,29^. Among pearl oysters of the genus, *P. margaritifera* is a good model for phenotypic colour variation studies, as it displays the largest range of pearl colours, reflected by the large diversity of inner shell colour phenotypes, in comparison to its two competing species *P. maxima* and *P. fucata*^3^. The green and yellow inner shell colour phenotypes have been studied recently and are known to also depend on rearing/culture site^30^, with a colour “signature” at the archipelago scale in French Polynesia^7^.

At the micro-scale observation level, assessment of the pearl surface growth fronts by electron microscopy showed the smallest distance growth front for the middle mantle section, where maximum growth was observed and is characteristic of the *Pinctada* shell shape. Such fronts have been already observed at the growth surface of bivalve nacre^31,32^ and in cultured pearls from *P. margaritifera*^33^, without looking for any direct connection between the pearl surface and the shell zone adjacent to their position of origin. The dynamics of nacre assembly therefore vary according to shell zone, and thus impact pearl quality depending on where the *saibo* was excised from the mantle. Change in structural assembly was related to variation in optical and mechanical properties, and was consequently connected to pearl colour, a character known to arise from light interference within the nanocomposite structure of the aragonite tablets^34^. This contributes to explaining the relation between microstructure and pearl colour expression.

At the molecular phenotype level, gene expression analysis, based on a panel of genes encoding proteins implicated in the shell biomineralisation process, indicated for the first time significant differences among donor phenotypes. The green and yellow donor groups revealed clearly different patterns of expression in the *saibo* tissue, particularly for the genes related to aragonite formation (*MRNP34*, *MSI60*, *Pearlin* and *Pif177*). The proteins corresponding to *Pif177* and *MSI60* genes regulate growth, nucleation and the organization of the aragonite crystal^25,35,36^. Pearlin is the protein equivalent to the N14 protein previously identified in *P. maxima*^37^ and seems to be specifically involved in the formation of the nacreous layer and promotion of aragonite crystal nucleation^38^. Whatever the mantle section, the *saibo* from the green donor phenotype showed systematic overexpression of these four aragonite-related genes compared with the yellow variant. These results could correspond to the higher proportion of good quality pearls (A–C and D+ grades), or difference among pearl colour distribution observed using the green phenotype compared to the yellow one. Pearl grade and colour was mainly attributed to aragonite tablets nature and assembly, which composed nacre produced from the internal regions of the mantle when this was used as *saibo*. The marginal area of the mantle produced the outer shell layer, constructed from densely packed calcite prisms, was associated with low pearl grade^39,40^. This marginal mantle area was excluded from the *saibo* cutting process. The different pattern of aragonite gene expression discriminating the two donor phenotype groups could not be attributed to any *saibo* cutting artefacts. Indeed, calcite gene expression pattern was equivalent for both donor phenotype group and followed the same tendency, such as for *Prism14* gene expression.

From an aquaculture point of view, such a phenome study could be of benefit to the pearl industry. At the scale of the donor phenotype, the present study detected specific donor-expression level of biomineralisation genes, with regard to the diversity of the shell colouration^29^. An establishment of systematic donor-specific phenomes could then be an interesting tool for the prediction of more appropriate colour lines to be selected and/or propagated for specific pearl colour and/or quality production. At the scale of the mantle graft phenotype, the results clearly show that mantle tissue from the posterior and connection sections can be successfully used as *saibo* for pearl production, as the resulting pearl quality traits were comparable to those produced with the middle zone, which is the part commonly used on commercial pearl farms, as was the nucleus retention rate. Indeed, by using these sections, more *saibo* could be obtained from the same number of donors. As the supply of pearl oysters for producers in French Polynesia has always been wild collection, finding colourful donor oysters was always a prerequisite, and sometimes limiting, step before the grafting process. Frequency of colourful donor oysters has been studied recently^29^, revealing that such individuals are rare and dependent on spat collection site. It is therefore crucial to find enough donor oysters to supply grafters, who need from 400 to 700 *saibo* per day.

Our exploration of phenotype variation at the level of *saibo* position was the first conducted in the *Pinctada* genus. A single donor oyster individual exhibiting a particular shell colour phenotype could therefore produce multiple pearl phenotypes at the scale of the *saibo* unit, mirroring its original activity at the mantle position level. This intra-phenotypic variation could overlap with the inter-donor phenotype variation. Our results suggest that systematic study of multiple phenotypes across multiple biological function and scales would be important in future phenome studies in *P. margaritifera*. Increased sample sizes could potentially succeed in revealing robust genetic associations with pearl quality trait improvement that would benefit the pearl industry.

## Materials and Methods

### Animals

First generation hatchery-produced *P. margaritifera* were used as donor oysters for this study. These oyster families were issued from multi-parental crosses using highly coloured broodstock with green “C” (Fig. 1a) and yellow “L” (Fig. 1b) phenotypes (inner shell coloration), carried out at the Regahiga Pearl Farm and Hatchery company, located on Mangareva Island (Gambier archipelago, French Polynesia). To discern the inner shell colour for this set, the grafter used a speculum to gently pry open the oyster valves. Broodstock breeding, larval rearing and culture of this family were done as described previously^41^. At the age of 30 months post-hatching, 24 donor pearl oysters from the green (N = 12) and yellow (N = 12) phenotypes, were randomly selected from a sets of healthy animals with a mean (±SD) dorso-ventral measurement of 113.9 ± 8.7 mm. The entire list of the shell length corresponding to each donor can be found in Supplementary Table S1.

Wild *P. margaritifera* were collected as spat in the Mangareva Island lagoon (Gambier Archipelago, French Polynesia) to serve as recipients. Passive techniques were employed for catching spat using commercial collectors. Oysters were reared from the juvenile to adult stage as previously described^42^. The oysters were used in the grafting procedure once they were almost 20 months old, with a mean (±SD) dorso-ventral measurement of 76.30 ± 6.5 mm.

### Experimental graft

The grafting operation was conducted in October 2014, with all grafts performed by an expert from the Regahiga Pearl Farm and Hatchery company as previously^43^. The nuclei used for this purpose were made from the shells of freshwater mussels (1.8 BU size, equivalent to 5.45 mm diameter, 0.26 g weight; Imai Seikaku Co. Ltd., Japan). The thickness and hardness of the nacreous layers of these beads offer a specific gravity and thermal conductivity that make them particularly suitable for use as pearl nuclei^44^. The epithelial cells required for grafting *saibo* were excised from the entire mantle of the selected donor pearl oysters and include all the following sections: posterior, connection, middle and anterior. A total of 1798 grafts were performed over five days (865 grafts using the green phenotype and 933 grafts using the yellow phenotype). Supplementary Table S1 gives the number of grafts performed for each donor oyster and per mantle section. All the grafted oysters were checked for nucleus retention/rejection and mortality 45 days after the grafting operation, as previously described^42^. The oysters that had retained their nuclei were drilled and fixed onto chaplets (within chaplets, oysters were attached in pairs to a rope with a monofilament fishing line), which constituted the rearing system. All recipient oysters were individually labelled (attribution of a plastic label with a number) in such a way as to maintain the traceability between graft position and corresponding harvested pearls. Furthermore, the pearl oysters were regularly cleaned in order to remove biofouling (epibiota), which can hinder healthy oyster growth and pearl production.

### Pearl quality trait measurements

After approximately 20 months, the cultured pearls were harvested. The pearls were cleaned by ultrasonication in soapy water (hand washing) with a LEO 801 laboratory cleaner (2-L capacity, 80 W, 46 kHz); they were then rinsed in distilled water. Pearl size was assessed by measuring nacre thickness and nacre weight as previously described^28^. Shape, colour and grade were evaluated for all pearls by the same professional expert from the Poe O Rikitea association, according to Tahitian pearl auction classification categories^7^.

### Electron Microscopy

The structure of the pearl surface was observed by scanning electron microscopy (SEM) with a Hitachi Analytical Table Top SEM TM3030 at the electron microscopy facility of Université de la Polynésie Française, Tahiti. Before observation, the pearls were sawn at the point of their largest diameter so as to be positioned flat on the observation plate. Subsequently, the surfaces were metallized (Quorum Technologies, Q150R ES model) with a thin layer of gold (15 nm). This step eliminates any electromagnetic load that might interfere with observation. Observations were based on pictures taken at the pearl surface and particularly of the space between two layers of aragonite deposition (magnification 1000x, accelerating voltage 15 kV). The gap between layers of aragonite deposition was measured with post-acquisition image tools: we measured the gap between a finished deposition layer and the next or previous one (Fig. 3). Several locations on the surface of the pearl were thus measured for a statistical analysis of the averages.

### Gene expression analysis

Two donors among the twelve per phenotype were randomly sampled. For each donor (N = 4), all *saibo* pieces prepared from 1 valve were preserved in RNAlater (Qiagen) (50 mg/mL). The *saibo* obtained from the other valve were used in the graft processes and the corresponding pearl sacs were all sampled (at the same time as the pearls) and likewise preserved in RNAlater (Qiagen). For the pearl sac sampling, the gonads were first cut from the recipient. The gonad tissue was then removed with a surgical blade to leave only a thin (<0.5 mm) layer of tissue surrounding the pearl. At this point, only the pearl sac and the pearl remained. Next, an incision was made in the pearl sac and the pearl removed. The pearl sac was transferred into a 5.0-ml tube with RNAlater® until RNA extraction^45^. The samples were stored at −80 °C until RNA extraction. A total of 170 *saibo* and 120 pearl sacs were sampled.

Expression levels were measured by RT-PCR for a panel of nine genes involved in biomineralisation: four aragonite-related genes (*MRNP34*, *MSI60*, *Pearlin* and *Pif177*), four calcite-related genes (*Aspein*, *Shematrin 9*, *Prismalin-14* and *KRMP7*) and one involved in the formation of both nacreous aragonite and prismatic calcite (*Nacrein A1*). Two housekeeping genes were also measured, chosen based on their ubiquitous and constitutive expression pattern in *P. margaritifera* tissue: SAGE (SAGES: AGCCTAGTGTGGGGGTTGG/ SAGER: ACAGCGATGTACCCATTTCC) (called REF^36^) and GAPDH (GAPDHS: AGGCTTGATGACCACTGTCC/ GAPDHR: AGCCATTCCCGTCAACTTC)^46^. Primer sequences of the nine biomineralisation genes can be found in Supplementary Table S2.

After removing the RNAlater by pipetting and absorption, total cellular RNA was extracted from the individual graft tissue (pooled into 55 samples with respect to the different mantle positions studied, with 3–4 *saibo* caming from the same individual per extraction) or pearl sac samples (N = 120), using TRIzol® reagent (Life Technologies) according to the manufacturer’s recommendations. *Saibo* from connection zone were not used for this part of the study as only 3 to 6 pooled sample could be obtained. RNA was quantified using a NanoDrop ND-1000 spectrophotometer (NanoDrop Technologies, Inc.). Total RNA of each individual was then treated with DNAse I using a DNA-free Kit (Ambion). First, strand cDNA was synthesized from 500 ng total RNA using the Transcriptor First Strand cDNA Synthesis Kit (Roche) and a mix of poly (dT) and random hexamer primers. Real-Time PCR amplifications were carried out on a Roche Light Cycler® 480. The amplification reaction contained 5 μL LC 480 SYBR Green I Mast (Roche), 4 μL cDNA template, and 1 μL of primer (1 µM), in a final volume of 10 μL. Each run included a positive cDNA and a blank control for each primer pair. The run protocol was as follows: initial denaturation at 95 °C for 10 min followed by 40 cycles of denaturation at 95 °C for 30 s, annealing at 60 °C for 30 s and extension at 72 °C for 60 s. Lastly, the amplicon melting temperature curve was analysed using a melting curve program: 45–95 °C with a heating rate of 0.1 °C s^−1^ and continuous fluorescence measurement. All measurements were made in duplicate and all analyses were based on the Ct values of the PCR products.

Relative gene expression levels were calculated using the delta–delta method, normalized with two reference genes, to compare the relative expression results^47^ as follows: Relative expression_(target gene, sample x)_ = 2^−(ΔCt sample, sample x−ΔCt calibrator, sample x)^ = 2^−ΔΔCt^. Here, the ΔCt calibrator represents the mean of the ΔCt values obtained for the tested gene. The delta threshold cycle (ΔCt) is calculated by the difference in Ct for the target and reference genes. The relative stability of the GAPDH and SAGE combination was confirmed using NormFinder^48^. PCR efficiency (E) was estimated for each primer pair by determining the slopes of standard curves obtained from serial dilution analysis of a cDNA to ensure that E ranged from 90 to 110%.

### Statistical analysis

All analyses were performed using R© version 3.2.3 software (R foundation for Statistical Computing). The significance threshold was set at *p* ≤ 0.05 and the tests were two-sided. All measures are given as means and variability as standard deviations.

Pearl quality and retention differences according to *saibo* positions were tested, for qualitative parameters, using χ² tests and Fisher’s exact tests when an expected value <5 was found. When significant differences were detected, pairwise comparisons were made for proportions. For quantitative categories, normality and homoscedasticity were tested using Shapiro–Wilk test and Bartlett’s tests, respectively. Due to non-normality, Kruskal–Wallis tests were used to test differences between *saibo* positions. When differences were detected, post-hoc analyses were performed with Dunn tests and Bonferroni correction. Kruskal-Wallis tests were also used to test difference of gene expression levels between the phenotypes.

### Data availability statement

The authors declare that all datas are available.

## Electronic supplementary material

Supplementary Information
