# Supplementary material for: Phenome of pearl quality traits in the mollusc transplant model Pinctada margaritifera
Source: Sci Rep. 2018 Feb 1;8:2122. doi: 10.1038/s41598-018-20564-1 (PMC5794767; doi:10.1038/s41598-018-20564-1)
Supplement: Supplementary file 1 — Supplementary Information [file 41598_2018_20564_MOESM1_ESM.doc]

**Supplementary information**

**Title of the manuscript:**

**Phenome of pearl quality traits in the mollusc transplant model *Pinctada margaritifera*.**

**Full author list:**

Chin-Long KY*, Virgile QUILLIEN, Floriane BROUSTAL, Claude SOYEZ, Dominique DEVAUX

**Supplementary table S1**. Shell height (mm) and number of *saibo* used from each of the 24 *Pinctada margaritifera* donor oysters used for the experimental graft design, according to their colour phenotype and the position of the mantle graft (posterior, connection, middle or anterior).

|  | Donor # | 1 | 2 | 3 | 4 | 5 | 6 | 7 | 8 | 9 | 10 | 11 | 12 | Mean / N |
| --- | --- | --- | --- | --- | --- | --- | --- | --- | --- | --- | --- | --- | --- | --- |
| Yellow phenotype | Height (mm) | 119 | 113 | 121 | 109 | 108 | 106 | 107 | 100 | 132 | 120 | 118 | 109 | 113.5 |
|  | Posterior | 10 | 10 | 20 | 20 | 20 | 20 | 20 | 20 | 20 | 20 | 20 | 20 | 220 |
|  | Connection | 3 | 3 | 6 | 6 | 6 | 6 | 6 | 6 | 6 | 6 | 6 | 6 | 66 |
|  | Middle | 25 | 19 | 42 | 42 | 42 | 38 | 36 | 40 | 37 | 37 | 37 | 36 | 431 |
|  | Anterior | 12 | 10 | 21 | 20 | 21 | 20 | 18 | 20 | 18 | 19 | 19 | 18 | 216 |
| Green phenotype | Height (mm) | 121 | 114 | 122 | 114 | 113 | 104 | 100 | 111 | 101 | 126 | 125 | 121 | 114.3 |
|  | Posterior | 10 | 10 | 20 | 20 | 20 | 20 | 20 | 20 | 20 | 20 | 20 | 20 | 220 |
|  | Connection | 3 | 3 | 6 | 6 | 6 | 6 | 6 | 6 | 6 | 6 | 6 | 6 | 66 |
|  | Middle | 18 | 18 | 41 | 36 | 36 | 35 | 32 | 36 | 30 | 32 | 36 | 36 | 386 |
|  | Anterior | 9 | 9 | 21 | 18 | 18 | 18 | 16 | 18 | 14 | 16 | 18 | 18 | 193 |

**Supplementary table S2:** Set of forward and reverse primers used for the biomineralization gene expression analysis in *Pinctada margaritifera*.

|  | **Primer name** | **GenBank Accession Numbers** | **Forward primer (5’-3’)** | **Reverse primer (5’-3’)** |
| --- | --- | --- | --- | --- |
| Aragonite  formation | **MRNP34** | HQ625028 | GTATGATGGGAGGCTTTGGA | TTGTGCGTACAGCTGAGGAG |
|  | **MSI60** | SRX022139^a^ | TCAAGAGCAATGGTGCTAGG | GCAGAGCCCTTCAATAGACC |
|  | **Pearlin1** | DQ665305 | TACCGGCTGTGTTGCTACTG | CACAGGGTGTAATATCTGGAACC |
|  | **Pif177** | HE610401 | AGATTGAGGGCATAGCATGG | TGAGGCCGACTTTCTTGG |
| Aragonite & calcite formation | **Shem9** | ABO92761 | TGGTGGCGTAAGTACAGGTG | GGAAACTAAGGCACGTCCAC |
|  | **NacreinA1** | HQ896199 | CTCCATGCACAGACATGACC | GCCAGTAATACGGACCTTGG |
| Calcite formation | **Aspein** | SRX022139^a^ | TGAAGGGGATAGCCATTCTTC | ACTCGGTTCGGAAACAACTG |
|  | **KRMP7** | ABP57445 | GCCTTCACCACAGAAGGAAG | GCCGAATTTCTTCAGACACC |
|  | **Prism14** | HE610393 | CCGATACTTCCCTATCTACAATCG | CCTCCATAACCGAAAATTGG |
